# Human placental hematopoietic stem cell-derived natural killer cells (CYNK) recognize and eliminate influenza A virus-infected cells

**DOI:** 10.3389/fimmu.2022.900624

**Published:** 2022-10-20

**Authors:** Manojkumar Gunasekaran, Andrea Difiglia, John Fitzgerald, Robert Hariri, William van der Touw, Tanel Mahlakõiv

**Affiliations:** Celularity Inc., Florham Park, NJ, United States

**Keywords:** NK cells, virus, antiviral, immunotherapy, influenza, cell therapy

## Abstract

Influenza A virus (IAV) infections are a significant recurrent threat to public health and a significant burden on global economy, highlighting the need for developing more effective therapies. Natural killer (NK) cells play a pivotal role in the control of pulmonary IAV infection, however, little is known about the therapeutic potential of adoptively transferred NK cells for viral infections. Here, we investigated the antiviral activity of CYNK, human placental hematopoietic stem cell-derived NK cells, against IAV infection *in vitro*. Virus infection induced the expression of NK cell activating ligands on respiratory epithelial cells, resulting in enhanced recognition by CYNK cells. Upon co-culture with IAV-infected epithelial cells, CYNK exhibited elevated degranulation and increased production of IFN-γ, TNF-α and GM-CSF in a virus dose-dependent manner. Furthermore, CYNK showed virus dose-dependent cytotoxicity against IAV-infected cells. The antiviral activity of CYNK was mediated by NKp46 and NKG2D. Together, these data demonstrate that CYNK possesses potent antiviral function against IAV and warrant clinical investigations for adoptive NK cell therapies against viral infections.

## Introduction

Seasonal influenza viruses primarily target epithelial cells of the upper airways, and the infection is typically associated with a mild respiratory illness. However, occasionally, the virus can spread from the upper airways to the lower respiratory tract resulting in pneumonia and acute respiratory distress syndrome. Influenza virus pandemics, epidemics and sporadic outbreaks result in heightened morbidity and mortality, therefore implying a significant burden on global healthcare (1, 2). While vaccination remains the most effective means to prevent and control influenza virus infections, vaccines have limited efficacy and need seasonal redesign due to rapid antigenic evolution (3). While various antiviral drugs exist, drug-resistant viruses have emerged (4). Alternative broad-spectrum therapies are therefore urgently needed to control emerging virus strains.

Natural killer (NK) cells are innate lymphoid cells that play a key role in immune surveillance against tumorigenesis and infectious microorganisms. NK cells circulate the body probing the environment using an array of activating and inhibitory cell surface receptors that distinguish between healthy and stressed cells. NK cell activating receptors, including natural cytotoxicity receptors (NCRs) NKp30, NKp44, NKp46 and NKG2D, recognize cell surface stress ligands that are upregulated on transformed or infected cells. In addition, NCRs recognize pathogen-associated molecules, such as viral hemagglutinins (HA), on infected cells (5–7). Target cell recognition leads to NK cell activation and specific target eradication *via* “injection” of cytolytic granules, containing granzymes and perforin, or by death ligand induced apoptosis (8, 9). Activated NK cells also secrete a variety of cytokines and chemokines, including interferon gamma (IFN-γ), tumor-necrosis-factor alpha (TNF-α) and granulocyte-macrophage colony-stimulating factor (GM-CSF) that support target cell killing and shape the developing adaptive immune response (10–12).

Recent studies have demonstrated robust activation of NK cells during viral infections, regardless of the virus class (13) and that the depletion of NK cells aggravates viral pathogenesis in animal models (14–18). In humans, it was reported that the number of NK cells decreased upon seasonal IAV infection in peripheral blood (19) and NK cell lymphopenia in the peripheral blood and lung was associated with disease severity during the 2009 pandemic H1N1 infection (20, 21). In addition to direct cytolysis of infected cells, lung-resident NK cells produce a significant amount of IFN-γ (22) that promotes humoral and cell-mediated immunity and contributes to survival in influenza infection in mice (23, 24). Similarly, NK cell-derived TNF-α contributes to reduced influenza virus replication in lung epithelial cells (25–27). The many facets of NK activity in respiratory infections was reviewed by Culley et al. (28). The protective role of NK cells during viral infections suggests that boosting NK cell numbers or activity may provide a clinical benefit against IAV. We have developed CYNK-001, an off-the-shelf allogeneic NK cell population derived from human placental hematopoietic stem cells, that efficiently kills various tumor cell lines *in vitro* and *in vivo* (29, 30). While adoptive NK cell therapies, including CYNK-001, are being investigated in the clinical setting for the treatment of hematological and solid tumors, NK cell therapies and their potential benefit in infectious diseases has been little investigated.

Here, we took the first step to assess the antiviral function of CYNK (research grade CYNK-001) using an *in vitro* IAV infection model on a respiratory epithelial cell line. We demonstrate that CYNK recognize IAV-infected cells *via* highly expressed NK cell activating surface receptors and lyse IAV-infected epithelial cells.

## Materials and methods

### CYNK cell culture

CYNK cells are the research-grade counterpart of CYNK-001, a cryopreserved off-the-shelf placental hematopoietic stem cell-derived NK cell product manufactured under current good manufacturing practice standards (30). CYNK stem cells are generated by expanding and differentiating placental hematopoietic CD34^+^ stem/progenitor cells in a 35-day culture process in the presence of cytokines, including thrombopoietin, stem cell factor, FLT3 ligand, recombinant human interleukin (rhIL)-7, rhIL-15, and rhIL-2 as described in (31). CYNK are stored frozen in the gas phase of liquid nitrogen.

### *In vitro* characterization of CYNK

Frozen CYNK cells were thawed and washed in staining buffer (10% fetal bovine serum (FBS) in phosphate buffered saline (PBS, Gibco)). CYNK cells were stained with LIVE/DEAD Fixable Aqua Dead Cell Stain (Invitrogen) in PBS, followed by blocking in Purified Mouse IgG2a, κ Isotype Control (BD), Human Fc Block (BD) and BD Horizon Brilliant Stain Buffer (BD) in staining buffer. Cells were then stained with fluorophore-conjugated antibodies from BD, Miltenyi Biotec and Biolegend diluted in staining buffer according to manufacturers’ instructions. The following antibodies were used: CD226 (DNAM-1) - PE (Clone: DX11, BD), CD337 (NKp30) – BV421 (Clone: p30-15, BD), CD335 (NKp46) – BV650 (Clone: 9-E2, BD), CD56 (NCAM1) – AF700 (Clone: 5.1H11, BioLegend), CD3 - APC-Cy7 (Clone: SK7, BD), CD14 - APC-Cy7 (Clone: MφP9, BD), CD19 - APC-Cy7 (Clone: SJ25C1, BD), CD336 (NKp44) – BV510 (Clone: p44-8, BD), CD314 (NKG2D) – APC (Clone: BAT221, Miltenyi Biotec). Samples were acquired on Cytek Aurora flow cytometer (Cytek) and data analyzed on FlowJo Software (BD).

### Influenza A virus

Influenza virus A/Puerto Rico/8/34 (IAV PR8) H1N1 virus was obtained from ATCC.

### Virus infection and analysis of NK cell receptor ligand expression

Human alveolar basal epithelial adenocarcinoma cell line A549 (ATCC) were maintained in a humidified incubator at 37°C and 5% CO_2_. A549 were seeded at a concentration of 10^6^ cells per well in a 6-well plate in complete F-12K medium (supplemented with 10% FBS, 100 units/ml penicillin G, 100 μg/ml streptomycin) (Gibco). After 24 hours, the cells were washed with PBS and infected with IAV A/Puerto Rico/8/34 strain in serum-free Opti-MEM medium (Gibco) at various multiplicity of infection (MOI). Infection was performed at room temperature with gentle rocking for 1 hour, followed by replacement of the inoculum with complete F-12K medium, and the cells were returned to the incubator. Cells were stained and analyzed using flow cytometry after 24 or 48 hours of infection. A549 cells were surface labelled with fluorophore-conjugated antibodies: anti-ULBP1 – PE (clone: 170818, R&D Systems), anti-ULBP-2/5/6 - PE (clone: 165903, R&D Systems), anti-ULBP3 - PE (clone: 170818, R&D), anti-MICA/B - APC (clone: 6D4, Biolegend), CD95 (Fas) – FITC (clone: DX2, Biolegend), CD155 – PE-Cy7 (clone: TX56, Biolegend), CD112 (Nectin-2) – PE-Cy7 (clone: TX31, Biolegend), B7H6 - APC (clone: 875001, R&D Systems), CD261 (DR4) - APC (clone: DJR1, Biolegend), DR5 - PE (clone: DJR2-4 (7-8), Biolegend) or Fc-coupled recombinant human NK cell receptor proteins NKp30, NKp44, NKp46 and NKG2D (all from R&D systems), followed by anti-human-Fc – Alexa Fluor 647 (clone: HP6017, Biolegend). Intracellular IAV nucleoprotein (NP) was stained with anti-influenza NP – FITC (clone: D67J, Thermo Fisher Scientific) after fixation and permeabilization of surface-stained cells with Cytofix/Cytoperm (BD). Cells were analyzed using Cytek Aurora (Cytek) and the data were analyzed using FlowJo Software (BD).

### Degranulation and intracellular cytokine staining

A549 cells were seeded at 10^4^ cells per well in a 96-well plate in complete F-12K medium. PR8 infection was performed as described above. CYNK cells were added 24 hours post infection at an effector-to-target (E:T) ratio of 10:1 with anti-CD107a – BV786 (clone H4A3, BD) in assay buffer (RPMI (Gibco) with 10% FBS). After 1 hour, 2 μM monensin (Biolegend) and 3μg/ml Brefeldin A (BD) were added and incubated for 3 hours. Cells were stained with Live/Dead Fixable Aqua Stain (Thermo Fisher Scientific) and labelled with anti-CD56 (NCAM1) – Pe-Cy7 (clone: 5.1H11, Biolegend), anti-CD3 – APC-Cy7 (clone: sk7, BD), anti-CD14 – APC-Cy7 (clone: mΦp9, BD) and anti-CD19 – APC-Cy7 (clone: SJ25C1, BD). For intracellular cytokine staining, surface-stained cells were permeabilized using Cytofix/Cytoperm (BD) and cells were stained with anti-TNF-α - PE (clone: MAb11, BD) and anti-IFN-γ - APC (clone: B27, BD) antibodies. Cells were analyzed using Cytek Aurora (Cytek) and the data were analyzed using FlowJo Software (BD).

### Cytotoxicity assay

A549 cells were seeded at 9x10^3^ cells per well in 96-well electronic microtiter plates (Agilent) in complete F-12K medium. The next day cells were infected with PR8 and 3-day recovered CYNK cells were added at an effector-to-target (E:T) ratio of 5:1. Cytotoxicity was measured using the xCELLigence Real-Time Cell Analysis (RTCA) platform (Agilent). Cell index, indicating the impedance of electron flow caused by adherent cells was recorded real time. Percentage of cytolysis was calculated using the formula: (cell index of no effector–cell index of effector)/cell index of no effector×100.

### NK receptor blocking

NK cell receptors were blocked for 30 minutes at 4°C on CYNK cells with 10 µg/ml of anti-NKp30 (clone: P30-15, Biolegend), anti-NKp44 (clone: P44-8, Biolegend), anti-NKp46 (clone: 9E2, Biolegend) and/or anti-NKG2D (clone: 1D11, Biolegend). IgG1 (clone: MOPC21, Biolegend) was used as a control. CYNK cells were then co-cultured with PR8-infected A549 cells at an E:T ratio 10:1 for 24 hours in the presence of indicated antibodies.

### Cytokine and chemokine quantification

CYNK cells were co-cultured with PR8-infected A549 cells at an E:T ratio 10:1 for 24 hours and cytokines and chemokines were quantified using Milliplex MAP Human CD8+ T cell magnetic bead panel (Millipore). Samples acquired using with the FlexMap 3D platform and data analyzed using Belysa curve fitting software.

### Statistical analysis

GraphPad Prism (GraphPad Prism Software, Inc.) was used for statistical analysis. All experiments have been repeated at least twice. A representative data is shown is not indicated otherwise. Statistical significance was shown as *, P < 0.05; **, P < 0.01 and ***, P < 0.001, ****, P < 0.0001.

## Results

**Figure 1:**
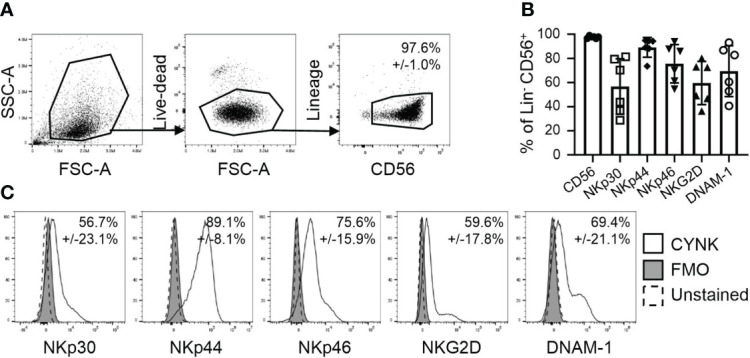
CYNK cells express NK cell activating receptors involved in virus recognition. **(A)** Representative flow cytometry dot plots demonstrating the gating strategy for the analysis of CYNK cells. Thawed CYNK cells were stained with fluorophore-conjugated antibodies recognizing indicated NK cell markers and analyzed by flow cytometry. Lineage included CD3, CD14 and CD19. CYNK are defined as live CD3^-^ CD14^-^ CD19^-^ CD56^+^ cells. **(B)** Proportion of indicated marker expression on CYNK cells. **(C)** Representative histograms of the expression of indicated NK activating cell receptors on CYNK cells. mean ± SD (n = 6) FMO - fluorescence minus one.

NK cells recognize virus-infected cells *via* specific receptor-ligand interactions. To explore the ability of CYNK to recognize virus-infected cells, we first analyzed the cell surface expression of NK cell activating receptors that are known to mediate the antiviral activity of NK cells. CYNK cells present with the nominal NK cell phenotype: a lack of expression of lineage markers CD3, CD14 and CD19, but high expression of CD56 (**Figure 1A**) and NK cell activating receptors NKp30, NKp44, NKp46, NKG2D and DNAM-1 (**Figures 1B, C**).

**Figure 2:**
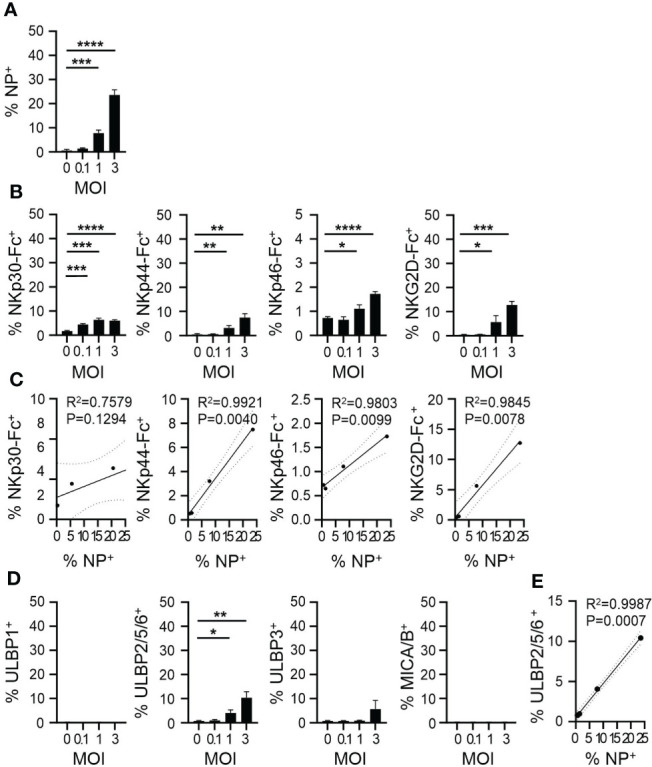
IAV upregulates NK cell ligand expression on infected cells. A549 cells were infected with influenza A virus strain A/Puerto Rico/8/34 (PR8) at MOI = 0, 0.1, 1 and 3 and cells were analyzed 24h post infection using flow cytometry. Expression of **(A)** intracellular viral nucleoprotein (NP) and **(B)** ligands of indicated NK cell activating receptors. **(C)** Linear regression analysis for the level of infection (x-axis: % NP^+^) and the level of NK receptor binding (y-axis). **(D)** Expression of indicated ligands of NKG2D. **(E)** Linear regression analysis for the level of infection (x-axis: % NP^+^) and the level of anti-ULBP2/5/6 binding (y-axis). mean ± SD (n ≥ 3). * indicates a statistically significant difference from IgG control in the experimental group. *P < 0.05, **P < 0.01, ***P < 0.001, ****P < 0.0001. Infected cells compared to non-infected cells (unpaired t-test). MOI, multiplicity of Infection.

To understand whether ligands for CYNK-expressed NK cell receptors are modulated by viral infection on target cells, we next established an *in vitro* infection model where a human lung epithelial cell line A549 is infected with IAV strain PR8/34 (H1N1) and analyzed the expression of NK cell activating receptor ligands on infected cells (**Supplementary Figure 1**). A549 cells were chosen as they are a common IAV *in vitro* infection model and are relatively resistant to NK cell killing even at high effector-to-target ratios (32). By 48h post infection, PR8 infection resulted in a significant cytopathic effect in A549 cells (**Supplementary Figures 1A-D**). As the NK cell receptors expressed on CYNK cells have multiple ligands, we used recombinant Fc-coupled NKp30, NKp44, NKp46 and NKG2D proteins to analyze their interaction with infected A549 cells. Virus infection significantly increased the binding of recombinant NKp30, NKp44, NKp46 and NKG2D proteins compared to non-infected cells (**Supplementary Figures 1E, F**). To further characterize the infection-induced ligands, we used antibodies against major known NK cell receptor ligands. PVR (CD155) and Nectin-2 (CD112), ligands for DNAM-1, were highly expressed but not modulated by infection. B7H6, a strong ligand for NKp30, was not expressed on A549 cells. Virus infection induced significant changes only in the expression of UL16 Binding Protein family members that activate NKG2D (**Supplementary Figure 1G**). To exclude that antibodies and proteins bound to the high proportion of dying cells at 48h post infection in a nonspecific manner, we titrated virus dose and analyzed virus nucleoprotein (NP) staining and NK cell receptor binding at 24h post infection (**Figure 2A**). IAV infection resulted in increased NP expression in a dose-dependent manner and induced up to 10% increase in NKp30, NKp44 and NKG2D ligand expression. NKp46 binding was increased by infection, however, to a lower extent (**Figure 2B**). Evaluation of the expression of NK cell receptor ligands on cells with increasing virus burden demonstrated a linear correlation between the level of virus infection and stress-ligand expression (**Figure 2C**). Of the major specific NK cell receptor ligands, increased staining of ULBP2/5/6 on infected cells was also noted at the 24h timepoint (**Figures 2D, E**). Together, the data suggest enhanced recognition of virus-infected cells by CYNK *via* specific receptor-ligand interactions.

**Figure 3:**
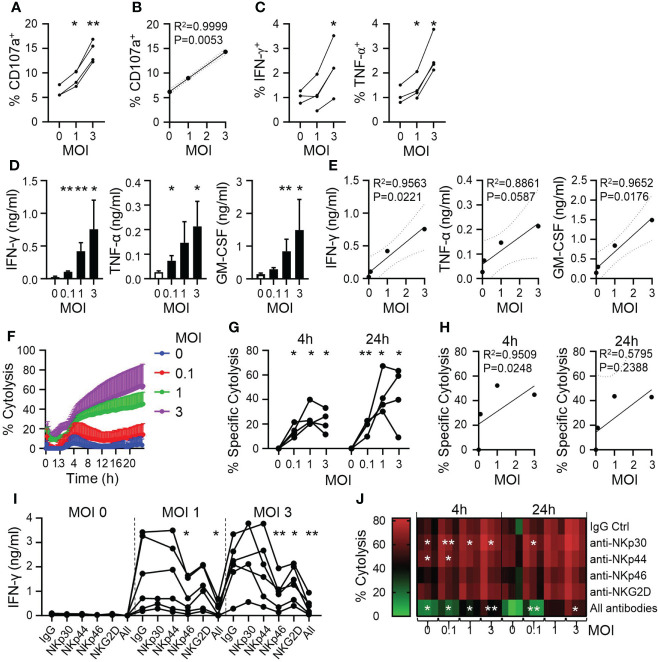
CYNK cells are activated by virus-infected cells in a dose-dependent manner. A549 cells were infected with influenza A virus strain A/Puerto Rico/8/34 (PR8) at the indicated MOI and CYNK were added 24h post infection at the E:T ratio of 5:1. **(A)** Expression of CD107a on CYNK cells (n = 4) **(B)** Linear regression analysis for virus dose (x-axis: MOI) and the level of CD107 expression (y-axis). **(C)** The proportion of CYNK cells positive for IFN-γ and TNF-α by intracellular cytokine staining after 4 hours of co-culture with virus-infected A549 cells. (n = 3-4) **(D)** Quantification of the indicated cytokines in cell culture supernatants collected after 24 hours of CYNK cells co-cultured with virus-infected A549 cells. **(E)** Linear regression analysis for virus dose (x-axis: MOI) and the level of secreted cytokine (y-axis). **(F, G)** Cytolysis of virus-infected A549 cells by CYNK cells analyzed using an impedance-based RTCA platform. **(F)** Cytolysis of virus-infected A549 by CYNK cells over 24 hours. **(G)** Specific cytolysis of virus-infected A549 cells after CYNK at 4h and 24h of co-culture. **(H)** Linear regression analysis for virus dose (x-axis: MOI) and the level of specific cytolysis at 4h or 24h (y-axis). **(I)** Quantification of IFN-γ in cell culture supernatants of CYNK cells co-cultured with infected A549 cells for 24h in the presence of indicated blocking antibodies: IgG, anti-NKp30, anti-NKp44, anti-NKp46, anti-NKG2D or all (anti-NKp30, anti-NKp44 anti-NKp46, anti-NKG2D). Data pooled from two individual experiments. **(J)** Cytolysis of virus-infected A549 cells by CYNK cells analyzed using an impedance-based RTCA platform in the presence of indicated antibodies or the combination of all (anti-NKp30, anti-NKp44 anti-NKp46, anti-NKG2D) at 4h and 24h. Paired t-test comparing treatment control (IgG) to blocking antibodies. * indicates a statistically significant difference from IgG control in the experimental group. mean ± SD (n ≥ 3 donors). *P < 0.05, **P < 0.01. E:T, effector-to-target ratio; MOI, multiplicity of infection.

To directly assess CYNK recognition of infected cells, we next analyzed CYNK activation upon co-culture with IAV-infected A549 cells. CYNK cells exhibited elevated cell surface staining of CD107a, a marker of degranulation that strongly correlated with the level of infection of target cells (**Figures 3A, B**). CYNK exposure to infected target cells also induced IFN-γ and TNF-α production, demonstrated by intracellular cytokine staining (**Figure 3C**). While cytokine staining was detected in a low proportion of CYNK, a significant amount of CYNK-secreted cytokines was detected in culture media (**Figure 3D**). The level of cytokine production by CYNK correlated with the virus dose used to infect A549 cells (**Figure 3E**). To understand whether virus infection sensitizes A549 cells to NK cell-mediated killing, we applied CYNK on virus-infected cells and monitored cell death in real time. Consistent with previous findings, virus infection in A549 cells induced cytotoxicity in CYNK against A549 cells in a virus dose-dependent manner (**Figures 3F–H**). While A549 is relatively resistant to NK cell-mediated killing, CYNK cell cytotoxicity against PR8-infected A549 cells increased from 5% at 0 MOI to 45% and 64% at MOI 1 and 3, respectively.

To analyze the involvement of specific NK cells receptors in the antiviral activity of CYNK, we blocked NK cell receptors on CYNK with antibodies in co-culture with virus-infected A549 cells. IFN-γ production was used as a sensitive readout for CYNK activation. NKG2D and NKp46 blocking resulted in significantly reduced production of IFN-γ, however, combining all tested antibodies further decreased IFN-γ production, suggesting a synergistic relationship between the NK cell receptors in CYNK activation (**Figure 3I**). In agreement with the latter, combined blocking of NK cell receptors resulted in decreased cytolysis of virus-infected cells. However, blocking of single receptors NKp30 and NKp44 mildly increased cytolysis of target cells, suggesting their potential inhibitory role on CYNK activity in this model (**Figure 3J** and **Supplementary Figure 2**).

Altogether, the data demonstrate that CYNK recognize infection-induced stress ligands on virus infected cells *via* specific receptor-ligand interactions, resulting in CYNK degranulation, cytokine production and target cell cytolysis.

## Discussion

Over the past decades, considerable progress has been made in the development of adoptive NK cell therapies for the treatment of cancer (33). First indications of a therapeutic potential of an NK cell therapy came from a trial where allogeneic NK cells were used to treat acute myeloid leukemia (AML) (34). Allogeneic culture-expanded or stem cell-derived NK cells have since been investigated in patients with various hematological or solid tumors (33, 35). CYNK-001, a cryopreserved allogeneic placental stem cell-derived NK cell therapy, is currently being investigated for various cancer indications: Phase I study in AML (NCT04310592), Phase I/II study in multiple myeloma (NCT04309084) and Phase I study in glioblastoma multiforme (NCT04489420). As per CYNK-001 Investigator’s Brochure, the initial clinical study data established CYNK-001 to be safe and well-tolerated in cancer patients. Due to its broad activating receptor expression profile, CYNK-001 recognize and lyse an array of tumor cells *in vitro* and the therapy is expected to eliminate tumor cells in patients with different tumor types (29, 30).

In addition to the potent anti-tumor effect, NK cells are critical for the innate immune response against various viral infections through cytolytic elimination of infected cells, however, NK cells are little investigated as a potential therapeutic for infectious diseases (13, 14, 36–38). Here, we analyzed the antiviral properties of CYNK in an *in vitro* influenza A virus (IAV) infection model. We showed that infection of a human respiratory epithelial cell line increased the expression of different stress ligands in a virus dose-dependent manner. These stress ligands are well established targets of activating receptors NKp30, NKp44, NKp46 and NKG2D on CYNK cells. Analysis of receptor involvement in target recognition and CYNK activation using blocking antibodies revealed that NKp46 and NKG2D contributed specifically to cytokine production. In the cytolysis model, only combined blocking of all analyzed receptors resulted in reduced target cell cytolysis. Individual blocking of receptors revealed, that NKp30 and NKp44 might play an opposing role as blocking these receptors increased the cytolytic response, suggesting that a synergistic role of NKG2D and NKp46 resulted mounting a cytolytic response. This recognition pattern agrees with earlier publications highlighting the dominant role of NKp46 in the recognition of virus-infected cells (5, 36, 38). NKG2D is another key regulator of NK cell responsiveness as its ligands are common markers of cell stress upon transformation or infection (9). CYNK has high expression of NKp44, a unique property compared to peripheral blood NK cells which have low or undetectable expression of NKp44 (29, 30, 39, 40). While NKp44 was shown to interact with viral HA and neuraminidase proteins resulting in target lysis, it could also behave as an inhibitory receptor, as seen here, due to an immunoreceptor tyrosine-based inhibitory motif (ITIM) in its cytoplasmic tail (41, 42). Several activating cellular ligands have been identified for NKp30, including B7H6, however, NKp30 interaction with viral molecules such as poxviral HA and human cytomegalovirus pp65 protein reduce cytolytic activity of NK cells (43–45). To date, no role has been identified for NKp30 on NK cells in influenza virus models. Our data suggests that NKp30 might inhibit cytolysis against IAV-infected A549 cells. Using recombinant NK cell receptors, we showed linear correlation between the level of infection and stress ligand expression on target cells. Furthermore, we established strong correlation between the level of target cell infection and CYNK cell responsiveness (degranulation, cytotoxicity and cytokine release), demonstrating a dominance of CYNK-activating signals on infected cells.

CYNK cell activation resulted in high secretion of immunomodulatory cytokines IFN-γ and TNF-α that directly participate in the innate response against various infectious agents but also promote the development of adaptive immunity (46–48). These pro-inflammatory cytokines, however, are also called a double-edged sword - their expression must be tightly controlled in space and time to balance the antiviral function and immunopathology. Excessive release of IFN-γ can result in excessive inflammation and tissue injury (49, 50).

To further understand the antiviral role of CYNK in IAV infection, we recently evaluated CYNK in an IAV-induced acute lung injury model in the mouse (51). The model requires the use of immunocompetent animals that are known to reject allogeneic human cells (CYNK). Furthermore, stress ligands on mouse cells might not be recognized by human NK cell receptors on CYNK. While the model is not optimal, CYNK administration improved clinical symptoms and reduced inflammation in infected mice. Our *in vitro* data, together with the *in vivo* findings, demonstrate a potent antiviral role for CYNK cells.

The significance of NK cells in the control of viral infections has been highlighted by multiple animal models where NK cells were deficient or defective, and in human NK cell deficiencies (52). All of these conditions correlate with increased susceptibility to viral infections and enhanced virus replication. A decrease in NK cell numbers and activity is a hallmark of IAV infection and coronavirus disease 2019 (COVID-19) (19–21, 53), suggesting that boosting endogenous immunity with adoptive transfer of highly activated NK cells, might offer a clinical benefit by containing the virus. CYNK-001 is an off-the-shelf product that is safe in humans and may offer a benefit to patients with viral infections, including IAV and COVID-19.

Together, these studies provide a strong argument for further investigation of NK cells in the context of virus infections and suggests that CYNK-001 could be developed as a treatment for infectious disease for patients with limited treatment options.

## Data availability statement

The original contributions presented in the study are included in the article/**Supplementary Material**. Further inquiries can be directed to the corresponding author.

## Author contributions

RH, WT and TM conceptualized the study. RH acquired funding. WT, RH and TM managed the study and provided supervision. MG, AD, JF and TM conducted experiments and analyzed the data. MG and TM prepared the first draft of the manuscript. All authors contributed to reviewing and editing of the manuscript and approved the final version.

## Conflict of interest

All authors are employed by Celularity Inc.

## Publisher’s note

All claims expressed in this article are solely those of the authors and do not necessarily represent those of their affiliated organizations, or those of the publisher, the editors and the reviewers. Any product that may be evaluated in this article, or claim that may be made by its manufacturer, is not guaranteed or endorsed by the publisher.

## Supplementary material

Supplementary Figure 1 IAV increases NK cell ligand expression on virus-infected cells. A549 cells were infected with influenza A virus A/Puerto Rico/8/34 strain (PR8) at MOI of 1, followed by analysis at 48 h post infection. **(A)** Representative images of phenotypic changes in PR8-infected A549 cells. **(B)** Proportion of live A549 cells post infection analyzed by flow cytometry. **(C)** A representative histogram demonstrating the proportion of viral nucleoprotein (NP) staining in infected cells. **(D)** Proportion of NP expression among PR8-infected infected cells. **(E)** Representative histograms demonstrating the binding of Fc-coupled recombinant NK cell receptor proteins to virus-infected cells (a readout for the expression of respective receptor ligands). **(F)** Quantification of Fc-coupled recombinant NK cell receptor protein binding to infected cells and Mean Fluorescence Intensity (MFI) of NK cell receptor ligand staining by Fc-coupled recombinant NK cell receptor proteins. mean ± SD (n = 3). **(G)** Quantification of the expression of the indicated NK cell receptor ligands on infected A549 cells and Mean Fluorescence Intensity (MFI) for the specific ligand staining. mean ± SD (n = 3). mean ± SD (n = 3). * indicates a statistically significant difference from IgG control in the experimental group. *P < 0.05, **P < 0.01, ***P < 0.001, ****P < 0.0001. Ctrl compared to CYNK (un-paired, t-test). MOI, multiplicity of Infection; Ctrl, control.

Supplementary Figure 2 Identification of CYNK cell receptors mediating lysis of virus-infected cells. A549 cells were infected with influenza A virus strain A/Puerto Rico/8/34 (PR8) at the indicated MOI and CYNK were added 24h post infection at the E:T ratio of 5:1. **(A)** Cytolysis of virus-infected A549 by CYNK cells in the presence of indicated individual blocking antibodies or the combination of all (anti-NKp30, anti-NKp44 anti-NKp46, anti-NKG2D) over 24 hours. Analysis performed using an impedance-based RTCA platform. **(B)** Cytolysis data from **(C)** graphed for 4h and 24h. Paired t-test comparing control (IgG) to blocking antibodies. * indicates a statistically significant difference from IgG control in the experimental group. mean ± SD (n≥3 donors). *P < 0.05, **P < 0.01. E:T, effector-to-target ratio; MOI, multiplicity of infection.
