# Supplementary figures and images for: Human placental hematopoietic stem cell-derived natural killer cells (CYNK) recognize and eliminate influenza A virus-infected cells
Source: Front Immunol. 2022 Oct 20;13:900624. doi: 10.3389/fimmu.2022.900624 (PMC9632418; doi:10.3389/fimmu.2022.900624)

Supplementary Figure 1

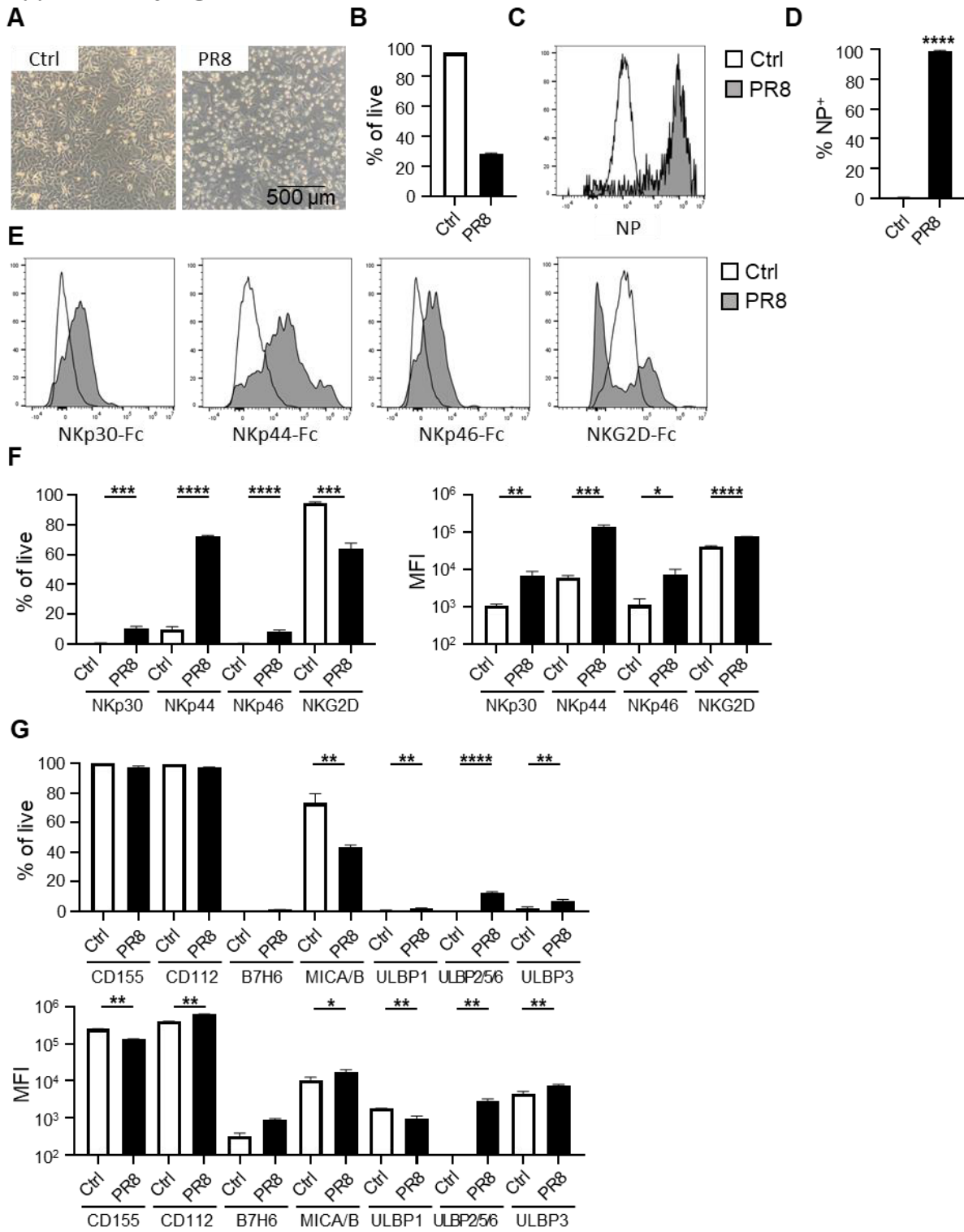

Supplementary Figure 2

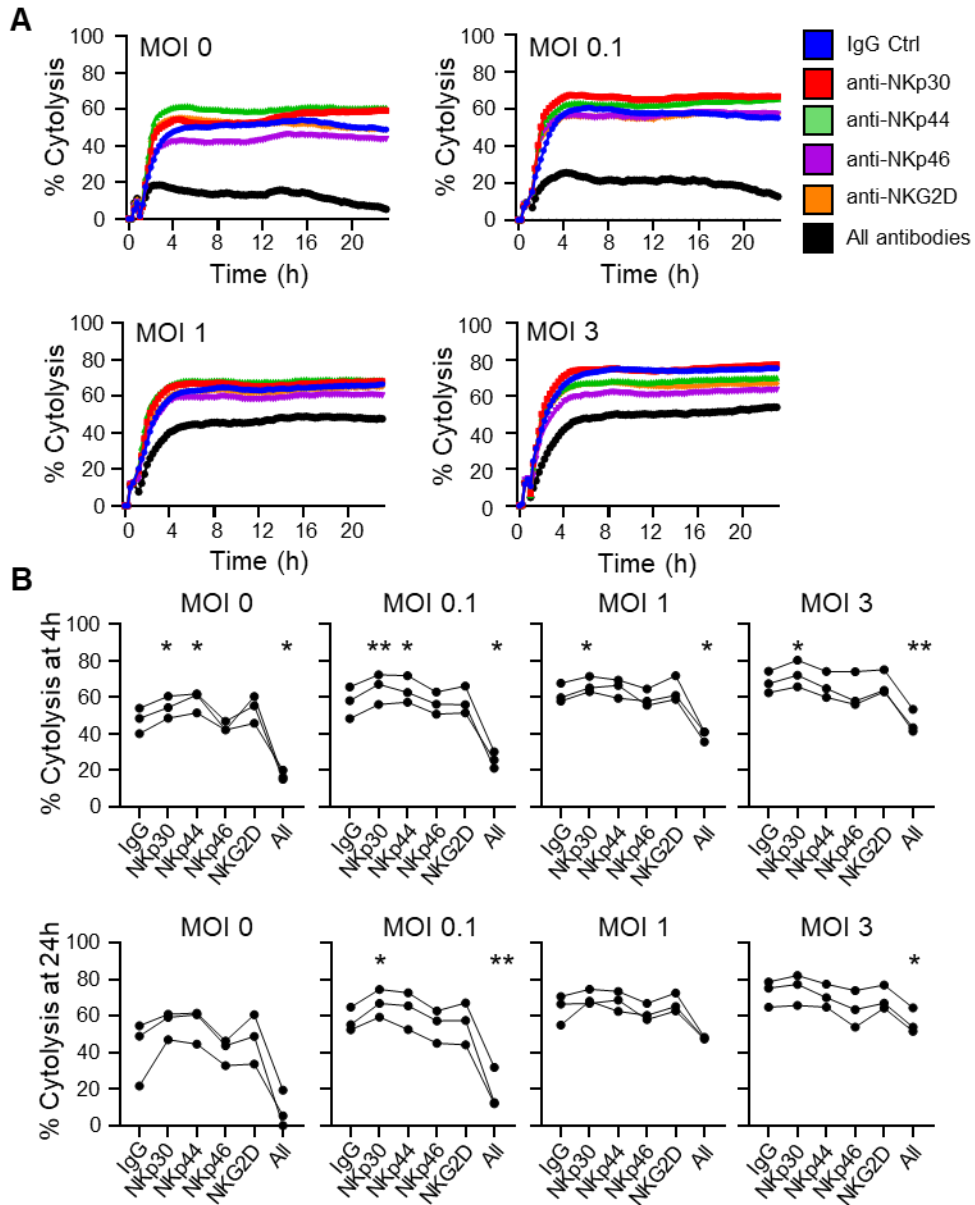

Supplement: Supplementary Figure 1 — IAV increases NK cell ligand expression on virus-infected cells. A549 cells were infected with influenza A virus A/Puerto Rico/8/34 strain (PR8) at MOI of 1, followed by analysis at 48 h post infection. (A) Representative images of phenotypic changes in PR8-infected A549 cells. (B) Proportion of live A549 cells post infection analyzed by flow cytometry. (C) A representative histogram demonstrating the proportion of viral nucleoprotein (NP) staining in infected cells. (D) Proportion of NP expression among PR8-infected infected cells. (E) Representative histograms demonstrating the binding of Fc-coupled recombinant NK cell receptor proteins to virus-infected cells (a readout for the expression of respective receptor ligands). (F) Quantification of Fc-coupled recombinant NK cell receptor protein binding to infected cells and Mean Fluorescence Intensity (MFI) of NK cell receptor ligand staining by Fc-coupled recombinant NK cell receptor proteins. mean ± SD (n = 3). (G) Quantification of the expression of the indicated NK cell receptor ligands on infected A549 cells and Mean Fluorescence Intensity (MFI) for the specific ligand staining. mean ± SD (n = 3). mean ± SD (n = 3). * indicates a statistically significant difference from IgG control in the experimental group. *P < 0.05, **P < 0.01, ***P < 0.001, ****P < 0.0001. Ctrl compared to CYNK (un-paired, t-test). MOI, multiplicity of Infection; Ctrl, control. [file Image_1.pdf]
